# Insights into the Role of Neutrophils and Neutrophil Extracellular Traps in Causing Cardiovascular Complications in Patients with COVID-19: A Systematic Review

**DOI:** 10.3390/jcm11092460

**Published:** 2022-04-27

**Authors:** Francesco Nappi, Francesca Bellomo, Sanjeet Singh Avtaar Singh

**Affiliations:** 1 Department of Cardiac Surgery, Centre Cardiologique du Nord, 93200 Saint-Denis, France; 2 Department of Clinical and Experimental Medicine, University of Messina, 98122 Messina, Italy; bellomofrancesca92@gmail.com; 3 Department of Cardiothoracic Surgery, Aberdeen Royal Infirmary, Aberdeen AB25 2ZN, UK; sanjeetsinghtoor@gmail.com

**Keywords:** SARS-CoV-2 infection, COVID-19, coronary artery thrombosis, neutrophil extracellular traps (NETs)

## Abstract

Background: The coronavirus disease 2019 (COVID-19) pandemic caused by the SARS-CoV-2 virus has resulted in significant mortality and burdening of healthcare resources. While initially noted as a pulmonary pathology, subsequent studies later identified cardiovascular involvement with high mortalities reported in specific cohorts of patients. While cardiovascular comorbidities were identified early on, the exact manifestation and etiopathology of the infection remained elusive. This systematic review aims to investigate the role of inflammatory pathways, highlighting several culprits including neutrophil extracellular traps (NETs) which have since been extensively investigated. Method: A search was conducted using three databases (MEDLINE; MEDLINE In-Process & Other Non-Indexed Citations and EMBASE). Data from randomized controlled trials (RCT), prospective series, meta-analyses, and unmatched observational studies were considered for the processing of the algorithm and treatment of inflammatory response during SARS-CoV-2 infection. Studies without the SARS-CoV-2 Infection period and case reports were excluded. Results: A total of 47 studies were included in this study. The role of the acute inflammatory response in the propagation of the systemic inflammatory sequelae of the disease plays a major part in determining outcomes. Some of the mechanisms of activation of these pathways have been highlighted in previous studies and are highlighted. Conclusion: NETs play a pivotal role in the pathogenesis of the inflammatory response. Despite moving into the endemic phase of the disease in most countries, COVID-19 remains an entity that has not been fully understood with long-term effects remaining uncertain and requiring ongoing monitoring and research.

## 1. Introduction

Since the first outbreak of the severe acute respiratory syndrome-coronavirus-2 infection (SARS-CoV-2), patients who developed coronavirus disease 2019 (COVID-19) frequently had cardiovascular involvement [1]. The myocardial injury was associated with high levels of troponin, especially among hospitalised COVID-19 patients [2]. However, the myocardial damage revealed by the increase in biomarkers was confirmed by echocardiography, which noted damage in 70% of hospitalized patients [3]. Therefore, cardiac involvement during COVID-19 was a truly probable event, despite the primary manifestation of disease within the lungs. Unfavourable outcome of the disease is likely in these subjects, which was immediately reported as sequelae of this complication [4]. Given these significant reports, the scientists’ attention has focused on two main clinical-pathological entities.

First, it must be emphasized that only a few patients with COVID-19 have experienced fulminant myocarditis, suggesting that this complication is rare [5,6]. In the small number of cases in which clinically suspected myocarditis was diagnosed, infection with SARS-CoV-2 was associated with cardiac inflammation [7].

Second, myocardial ischaemia, attributable to thrombotic coronary obstruction, appears to be the most likely event at the origin of myocardial damage, however, other causes such as heart failure, pulmonary embolism, tachycardia, and sepsis cannot be excluded [8]. Acute cardiac injury occurs in patients who experienced severe COVID-19 and confers serious complications and patient mortality [9].

We know that SARS-CoV-2, in addition to causing severe acute respiratory syndrome, has been shown to predispose infected patients to thrombotic disease with the involvement of arterial and venous vascular districts [10]. This complication is assumed to be secondary to uncontrolled inflammatory process, platelet activation, endothelial dysfunction, and marked stasis [11].

Recently the attention of several reports has suggested that in patients with severe organ dysfunction, SARS-CoV-2 infection is associated with excessive formation of neutrophil extracellular traps (NETs) with consequent vascular damage [12]. Furthermore, the autopsies performed in patients with unfavorable outcomes revealed a vascular mechanical obstruction due to the aggregates of NET, identifying in this process a central moment that is decisive in the complex pathogenesis of COVID-19 [12,13].

**Figure 1:**
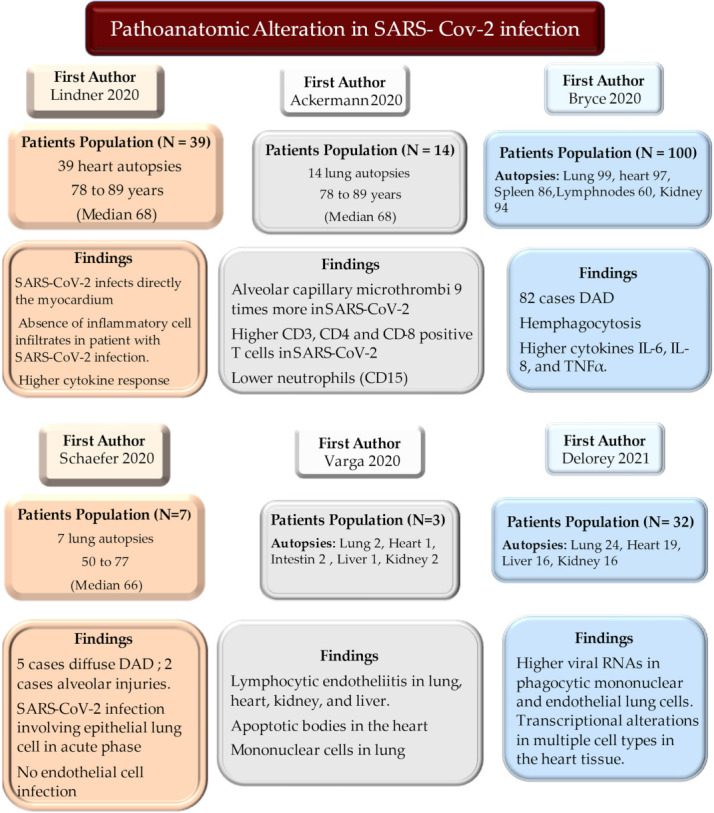
Autopsies substantially contributed to unveiling many unsolved aspects relating to the pathogenesis revealing the role of mononuclear cell infiltration leading to increased cytokine expression in patients who died with single or multi-failure organ pathologies. Abbreviations; DAD, diffuse alveolar damage; IL: interleukine; SARS-CoV-2, severe acute respiratory syndrome coronavirus 2; RNA, ribonucleic acid; TNF, tumor necrosis factor.

The role of mononuclear cells is decisive, either during myocarditis or coronary thrombosis due to activation of NETs, thus unearthing the controversial presence of SARS-CoV-2 in myocardial tissue and its potential for replication within the heart structures (cells and extracellular matrix). However, the role of mononuclear cell infiltration that induces increased cytokine expression remains elusive, both in patients who died without the signs of clinically evident myocarditis and in those who died in the absence of ST-elevation that characterized the myocardial ischemia due to coronary obstruction [13,14].Given the critical clinical context in which COVID-19 often occurs, burdened by a high percentage of deaths, the autopsies have contributed to unveiling many unsolved aspects related to its pathogenesis [13,15,16,17,18,19]. To foster a wider knowledge of mechanisms leading to myocardial injury and to provide a guide for clinicians, we herein debate the ongoing evidence basis on the role of NETs and propose an evidence-based algorithm for the prevention and control of inflammatory response during COVID-19 infections, Figure 1.

## 2. Search Method and Systematic Literature Review

**Figure 2:**
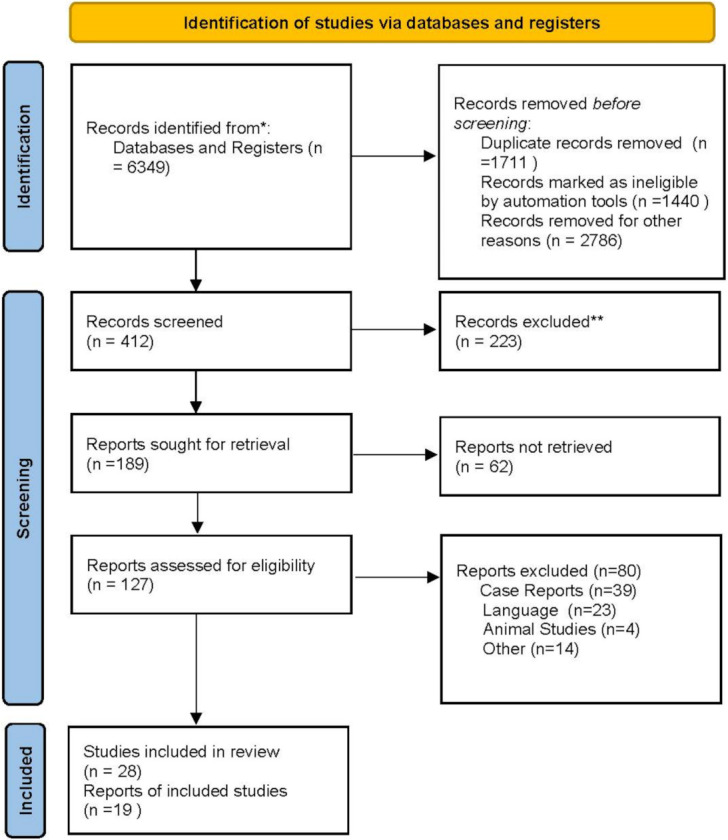
Prisma FloW Chart 2020 allowed to reach 47 determinant publications for the systematic review. * Search database; ** excluded for no meet criteria.

In December 2021, databases (MEDLINE; MEDLINE In-Process & Other Non-Indexed Citations and EMBASE) were searched using the terms “SARS-CoV-2”, “COVID-19”, “myocarditis”, “myocardial ischemia” and “neutrophil extracellular traps”, coupled with “inflammation”, mononuclear cell”, “neutrophil cell”, “cytokine”, “cytokine storm”. For this study, abstracts of included manuscripts were assessed and correlated. The present review focuses on data from randomized controlled trials (RCT), prospective series, meta-analyses, and unmatched observational studies that were considered for the processing of the algorithm and treatment of inflammatory response during SARS-CoV-2 infection. Data were extracted from the main publication, and searches were performed by two independent researchers (F.B, SSAS using blind method). A third independent reviewer estimated pertinence (FN). No funding was received for this study. The review was not formally registered. The protocol was not prepared. The authors have no conflicts of interest to declare. Prisma flow diagram for systematic review and Prisma checklist are reported in Figure 2, Table 1 and Table 2.

## 3. Results

### 3.1. Description of the Included Studies and of the Population

A total of 6349 studies was reported of which 412 studies were screened. 47 of these met the inclusion criteria and were included in the final systematic review (Flowchart). A total of 28 studies were international and/or multicentre of which 9 were from China and 6 from the USA; 3 prospective and 1 randomized multicenter clinical trial included approximately 116 countries. Most of the single-center studies were from China (Table 1). The number of patients in the individual studies ranged from 3 to 153,760. In clinical studies 99 to 153,760, autopsy studies 3 to 411, immune profile studies 7 to 349, and thromboembolism studies 12 to 1144 (Table 1).

### 3.2. Evidence from Neutrophil Deployment: Target Organs and Mechanism of Action

Pre-existing cardiovascular disease (CVD) represents a significant risk factor in patients with SARS-CoV-2 infection who develop COVID-19. Once SARS-CoV-2 infects the myocardium, it can cause direct or indirect damage. Likewise, in these patients, outcomes are worse than in patients without CVD [55]. A specific role favoring the post-inflammatory injury is played by neutrophils that work as major representative cells of the innate immune system. The formation of extracellular neutrophil traps (NETs) is included among the multiple functions that neutrophils perform [56]. Neutrophil extracellular traps (NETs) are released by neutrophils to counter infections through the formation of extracellular webs of chromatin, oxidizing enzymes, and microbiocidal proteins [56].

#### 3.2.1. COVID-19 and Inflammation

The first phase of infection with the development of COVID-19 begins with exposure to micro-droplets found in the exhalations of infected individuals. SARS-CoV-2 subsequently progresses to the bronchioles and alveolar spaces [57], where it is trapped in host cells (e.g., endothelial, epithelial, and smooth muscle cells) using a metallopeptidase available on the cell surface as the gateway, which is represented by the angiotensin-converting enzyme 2 (ACE2) [20,58,59,60]. We know that reaching the lung, SARS-CoV-2 infects alveolar cells (type I and II pneumocytes and alveolar macrophages) triggering intracellular replication mechanism in lung tissue. First early defense against the viral attacher is constituted by the production of type I and III interferons (IFN) which therefore have the role of inducing a premature defense mechanism to ensure the functional integrity of alveolar cells [57]. Recently, investigators disclosed an inadequate expression of these cytokines, other than the upregulation of the expression of chemokines and interleukins [61,62]. In normal human bronchial epithelial cell cultures (NHBE), an inhomogeneous profile affects cytokines. IFN deficiency is countered by an elevated expression of CCL20, CXC-type chemokines, IL-1β, IL-6, and tumor necrosis factor (TNF) [63,64,65]. In cell cultures exposed to SARS-CoV-2, the lack of IFN types I and III was evident, as despite susceptibility to the antiviral effect of IFN, SARS-CoV-2 retained the ability to inhibit its induction [62,63,64,65].

Likewise, SARS-CoV-2 positivity in cardiac tissue as well as in CD3+, CD45+ and CD68+ cells in myocardium and gene expression of tumor necrosis growth factor α, interferon γ, chemokine ligand 5, as well as interleukin-6, -8 and -18 were found in cell cultures from autopsy findings of patients who died from COVID-19 [13]. Regarding the production of interferon, it seems clear that the reduction may derive at least in part from the triggering of a mechanism that blocks the activation of the IFN signaling pathway. This process can occur at an early stage after the nuclear transport of the interferon regulatory factors (IRF) [66].

We learned that the different inflammation pattern in the involved tissues was related to the recruitment of leukocytes. This is an imprint of authentication of the inflammatory response which is firmly linked to the chemokine profile. Therefore, the inflammatory site can be affected by one cell type over another. This process depends on the profile of the chemokines that act as drivers, conditioning the different pathologies that characterize the SARS-CoV-2 infection [67]. The increased presence of monocytes/macrophages is due to the production of chemokine ligand 2 (CCL2) and CCL8 responsible for their recruitment while chemokine (C-X-C) ligand 16 (CXCL16) is a powerful chemoattractant for Natural Killer (NK) lymphocytes. Interleukine 8 (CXCL8) is the main chemoattractant of neutrophils whereas chemokine CXCL9 and CXCL10 can recruit T cells that recognize these molecules as specific chemoattractants [67].

A variation between moderate and severe COVID-19 was immediately noticeable and relies on the different immune characteristics of the patients. These features can change after ten days of infection whereby individuals with a trend towards worsening symptoms experience elevated levels of proinflammatory cytokines [21]. Furthermore, in the forms of COVID-19 marked by nefarious evolution, the dysregulation of the inflammatory response to the SARS-CoV-2 infection can be responsible for the cytokine storm syndrome [22,68]. Regarding heart involvement, although unusual cases of COVID-19 fulminant viral myocarditis have been revealed, recent evidence has suggested that some individuals can exhibit direct damage to the myocardial tissue, albeit in small percentages of cases [13,14].

**Figure 3:**
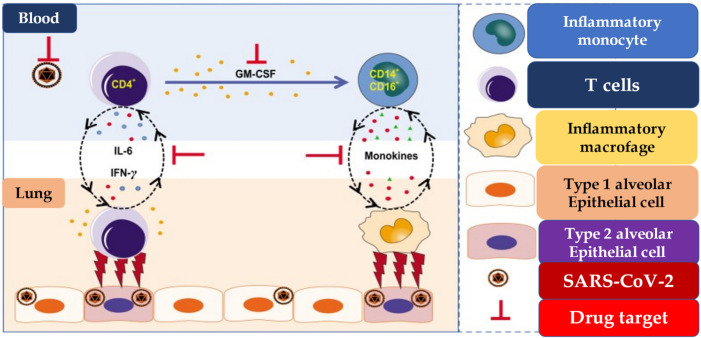
Pathogenic Th1 cells and inflammatory monocytes in severe COVID-19. Pathogenic CD4^+^ Th1 (GM-CSF^+^IFN-γ^+^) cells were rapidly activated to produce GM-CSF and other inflammatory cytokines to form a cascade signature of inflammatory monocytes (CD14^+^CD16^+^ with high expression of IL-6) and their progeny. These activated immune cells may enter the pulmonary circulation in large numbers and played an immune-damaging role in severe-pulmonary-syndrome patients. The monoclonal antibodies that target the GM-CSF or interleukin-6 receptor may potentially prevent or curb immunopathology caused by COVID-19. Abbreviations; GM-CSF, granulocyte-macrophage colony stimulating factor; IL, interleukine; IFN-γ, interféron gamma; SARS-CoV-2: severe acute respiratory syndrome-coronavirus-2.

The cytokine storm syndrome is distinguished by high levels of interleukins, TNF-α, G-CSF, monocyte chemoattractant protein-1 (MCP-1), and: macrophage inflammatory protein 1 (MIP-1α), which remain higher in patients needing admission to the intensive care unit (ICU) than in patients who require this degree of clinical observation [21,23,24]. Furthermore, several studies have revealed that the NOD-like receptor family, pyrin domain containing 3 (NLRP3) inflammasome, a multiprotein complex crucial for host defense, maintains a high level of activation in patients with COVID-19. It is important to underline that prolonged activation of NLRP3 leads to an increase in the levels of IL-1β and IL-18 which are associated with more severe forms of COVID-19 [25,69,70]. The cytokine environment orchestrates the recall of immune cells by activating the T helper 1 (Th1) response, which is configured as type-specific immune response involved in the inhibition of macrophage activation and stimulation of B cells to produce IgM, IgG1. The most important function of Th1 cells includes the production of IFN-γ, a signature cytokine that activates macrophages and DCs to present antigens to T lymphocytes. Th1 cells can also secrete tumor necrosis factor (TNF), lymphotoxin, and IL-2 which help to give a solid immunological response in the host. High levels of IL-6 production were recorded and reliant on the inflammatory monocytes’ activation as the distinct functions of Th1 cells in the severe form of COVID-19. This interaction supports the cytokine storm event [71], Figure 3.

Lindner et al. found high levels of IFN-γ and TNF in myocardiocytes of patients who died of COVID-19 suggesting that a Th1 response was elicited. Ref. [13] Huang et al. revealed that Th2 cytokine levels are detectable in patients with COVID-19 serum and their production can alter the response by modifying the Th1-inflammatory response [23]. Thus, as previously revealed by Lee et al. who reported that both inflammatory cytokine levels and a shift in Th1/Th2 balance worked as prognostic markers for hepatocellular carcinoma, ref. [72] the chemokine/cytokine environment coupled with the severe inflammatory response of the host may lead to potentially negative effects on the heart [13,14,55]. On the other hand, the environment where the chemokines/cytokines operate can constitute a possible target for the action of specific drugs used in the treatment of COVID-19 [73].

While awaiting the results from studies based on the pathoanatomical analysis of autopsy findings (heart, lung, kidney, and gastrointestinal system), which have demonstrated the occurrence of specific damage in the several tissues of deceased COVID-19 patients’, ref. [13,26,27,28,74] the first reports have documented the substantial variation in the rate of peripheral blood immune cells (PBMC) in COVID-19 patients’ [20,55]. Several convincing results have provided detailed answers on both the change in the percentage of cells of the immune response and the expression of HLA-DR genes [20,21,23,29,30,75]. Lucas et al. performed a longitudinal analysis from a large series of COVID-19 patients revealing an increased level of monocytes with a reduction of HLA-DR expression in the blood of infected individuals compared to that of the uninfected control cohort [23]. Evidence from other studies, involving patients with the severe form of COVID-19, disclosed a numerical reduction of B cells and NK cells associated with severe T-cell depletion. Instead, the neutrophil population recorded a considerable increase [21,23,29,30,75].

The increase in the rate of neutrophils varies with the worsening of clinical conditions and was generally observed after the seventh day from the onset of symptoms [31]. We recently reported a difference in the levels of immune response cells in the autopsy tissues of patients with poor outcomes [76].

#### 3.2.2. Neutrophils Activation: Crucial in SARS-CoV-2 Cardiac Infection

Xie et al. analyzed data from nearly 154,000 U.S. veterans infected with SARS-CoV-2 providing evidence on the long-term cardiovascular outcomes of COVID-19 [32]. Patients were monitored during the following year after recovering from the severe form of the disease and noted to have an increased risk of developing a higher rate of cardiovascular complications. These included cases of heart rhythm abnormalities, inflammation of the heart muscles, blood clots, strokes, myocardial infarctions, and heart failure. The most relevant data emerged at 12-months, showed that the cohort of patients with COVID-19 compared to the control cohort had been associated with an additional 45.29 incidents for every 1000 people evaluated of any prespecified cardiovascular outcome [32].

The major concern related to the increased risk of long-term cardiovascular outcomes was the development of a cardiac inflammatory reaction sustained by the neutrophilic reaction. Neutrophils represent the most abundant immune cells in human blood (50–70% of all leukocytes). Given their function to serve as fundamental cells in counteracting a large number of infections, neutrophils play a critical homeostatic role working in the context of chronic inflammatory diseases [77]. Although these polymorphonuclear cells and NETs have the distinctive role of arousing a well-defined immune response against bacterial or fungal infections, their function in the context of viral infections is not entirely clear, especially with the development of the necroinflammation phenomenon [78,79].

The acute clinical manifestations of COVID-19 have been well characterized by a systemic inflammation leading to the development of sequelae in several organ systems, including cardiovascular disorders [20,33]. We learned, from limited evidence, that neutrophils improve antiviral response by interconnection with various immune cell populations. While fulfilling their tasks, the following specific actions have been taken into consideration: virus internalization and killing mechanism, cytokine release, degranulation, oxidative burst, and neutrophil extracellular traps (NETs) formation [79,80]. This sequence of events can lead to a series of accidents which in the first phase of the disease affect the respiratory system, but can subsequently extend as a pan-systemic inflammation favoring the onset of many other sequelae, which include cardiovascular disorders, gastrointestinal disorders, malaise, fatigue, musculoskeletal pain, nervous and neurocognitive system disorders, mental health disorders, metabolic disorders, and anemia [33].

The association between the presence of elevated levels of neutrophils at the site of infection and the development of pulmonary disease associated with acute respiratory distress syndrome (ARDS) is very frequent and has been documented in both influenza virus infection and SARS-CoV-1 [81]. Using a bioinformatics analysis method, Hemmat et al. revealed that neutrophil activation and degranulation were extremely powerful processes during SARS-CoV infection [82]. Likewise, the recruitment of polymorphonuclear (PMN) cells have been reported as a crucial hinge-point in the host immune response to COVID-19 associated with critical illness. Again, neutrophilia has been used to gauge the severity of ARDS and poor outcomes in patients with COVID-19.

In patients who exhibit the severe form of COVID-19, abnormal blood clots were described in association with pulmonary embolisms in the lungs and deep vein thromboses localized to the peripheral arterial and venous vascular branches of the legs. Dysregulated clot assembly leads to strokes or heart attacks [34,35,83]. This event is promoted by the formation of autoantibodies [84] and it is supported by an alteration of the neutrophil-to-lymphocyte ratio (NLR), which is one of the most relevant clinical inflammatory biomarkers. The increased NLR correlates and forecasts severe illness, especially when it emerges in the early stage of SARS-CoV-2 infection [36,37,38,85].

Some pooled data [24,39,40,41,86,87] have suggested that the emergence of severe COVID-19 was related to higher levels of D-dimer and C-reactive protein (CRP) that arises after the augmentation in NLR in critically ill COVID-19 cases [39,86]. Likewise, the corroboration of some comorbidities such as diabetes and CVD [87] associated with the increasing of NLR has been reported as an independent risk factor for mortality in hospitalized patients [24,40,41]. In particular, Liu et al. observed that the presence of diabetes with higher NLR in patients with COVID-19 leads to a more severe clinical picture with a longer hospital stay [88]. The conclusion of the investigators supported the idea that sustained chronic inflammation may favor a more severe COVID-19 [40,89].

Wang et al. and Varim et al. independently reported that the involvement of PMN cells leading to a substantial change in neutrophil/CD4+ lymphocyte index (NCD4LR) and the neutrophil count to albumin ratio (NAR), thus accounting for worsening progression of COVID-19 [42,90]. The first study found that although the fluctuation of the NLR ratio is a very selective diagnostic index of increased inflammatory response in patients with COVID-19, during SARS-CoV-2 infection the NCD4LR was associated with negative conversion time (NCT). The investigators have suggested that patients who exhibit elevated NCD4LR have a poorer immune function and prolonged virus clearance [42], which may be due to early cardiac complications such as ST elevation myocardial infarction (STEMI) [14]. The second study revealed that the NAR biomarker could be considered a new predictor of mortality in COVID-19 patients [90].

It may be speculated that NCD4LR and NAR values may also be used as clinical markers for COVID-19 progression in association with NLR [24] in patients with coronary artery disease and arterial hypertension, in which an increase of neutrophils is not only reported in the bloodstream, but also in the lungs and in the heart [18]. In patients with CVD who succumbed to deterioration of clinical condition following COVID-19 diagnosis, histological analyses revealed an accumulation of inflammatory cells associated with endothelium, as well as apoptotic bodies in the heart [18].

We learned that neutrophil infiltration is an unfavorable factor in patients with cardiovascular complications, the latter behaving readily as a key threat in COVID-19 in association with lung disease [43,91]. However, the role of neutrophils must be evaluated in a more organic context that involves angiotensin-converting enzyme 2 receptor (ACE2) and endothelial cells, since SARS-CoV-2 uses ACE2 as a gateway into the host. This receptor is expressed in several organs, including the heart, lung, kidneys, intestines, and endothelial cells [92]. Although PMN infiltrates are strongly associated with vascular derangements in COVID-19. However, whether this disequilibrium is due to endothelial cell involvement by the virus remains uncertain. Our current understanding suggests that human blood vessel organoids are directly infected by SARS-CoV-2 in vitro [93]. Varga et al. disclosed that circulatory failure due to myocardial infarction and ST-segment elevation complicated with right heart failure, cardiac arrest resulted in death were associated with PMN infiltration and lymphocytic endotheliitis in heart as well as lung, kidney, and liver with evidence of cell necrosis. The investigator pointed out that the emerged histological evidence of myocardial infarction was not associated with lymphocytic myocarditis. [18].

**Figure 4:**
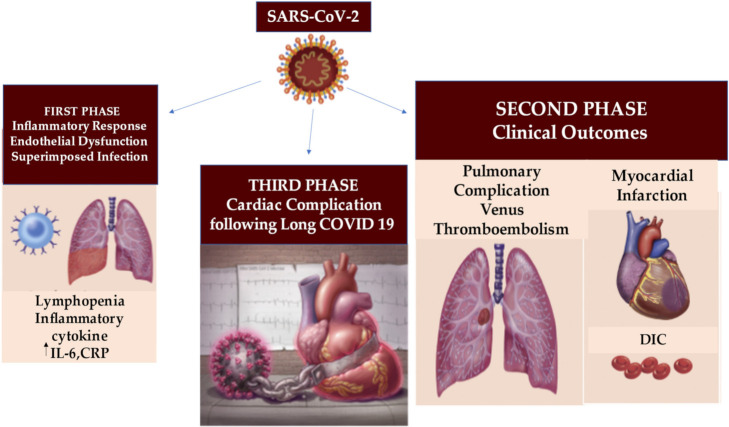
The acute clinical manifestations of COVID-19 are well characterized in the first and second phase, revealing an inflammatory response, endothelial dysfunction and overlapping infection that can evolve into thromboembolic and pulmonary complications, myocardial infarction and DIC. The third stage determines the COVID-19 heart condition after SARS-CoV-2 infection in which patients may reveal a range of increased cardiovascular risks. Abbreviations; CRP, C-reactive protein; DIC; disseminated intravascular coagulation. Other abbreviations in the previous figures. ↑, increase.

Another intriguing point is the discovery of immature phenotype and/or dysfunctional mature neutrophils that have been reported in the severe form of COVID-19 [94,95]. These studies indicate that increased infiltration of immature and/or dysfunctional neutrophils leads to an imbalance of the immune response of the lungs in severe cases of COVID-19, [96,97] in which cardiovascular atherosclerosis involvement and endothelitis occur [14,18], Figure 4.

#### 3.2.3. Neutrophils Extracellular Traps in COVID-19: The Hypothesis Takes Shape toward a Defined Role

Neutrophils extracellular traps are formed after the activation of neutrophils. The first description of the Nets was provided by Brinkman et al. who gave a new impetus to the investigation domain of granulocytes [56,98].

The structure of NETs is provided by nuclear chromatin to which nuclear histones and granular antimicrobial proteins are aggregated. NETs behave as scaffolds and this specificity makes them key elements to imprison microbes. Pathogens such as bacteria, fungi, viruses, and protozoa are killed once trapped [56,98]. This process is finalized inside the DNA fibers, avoiding the spread of pathogens and facilitating the concentration of antimicrobial factors at the site of infection [98].

NETosis orchestrates the entire process that leads to the formation of NETs and delineates a specific type of cell death, different from necrosis and apoptosis. Several studies have ascertained a very distinct role of NETosis in various infectious and non-infectious pathology such as the involvement in autoimmune diseases, cancer, venous thromboembolism, atherosclerosis, diabetes, etc. [99,100,101].

Briefly, NETosis is a cell death program that takes place in several stages which include the translocation of enzymes from the granules to the nucleus which facilitates chromatin decondensation. Importantly, the rupture of the internal membranes is recorded with the subsequent cytolysis and the release of the NETs. It should be pointed out that the main characteristic of NETosis is the disintegration of both the nuclear and granular membranes, but the integrity of the plasma membrane is preserved. This is a biological behavior that differentiates it from apoptosis or necrosis. The disruption of the nuclear wrapper during NETosis leads to the mixing of nuclear and cytoplasmic material, the loss of internal membranes, and the disappearance of cytoplasmic organelles. In detail, this process is marked by the absence of the peculiar signs of apoptosis such as the production of membrane bubbles, exposure to phosphatidylserine, condensation of nuclear chromatin, and DNA fragmentation [56].

In NETosis the intracellular proteins escape from the cells as both the nuclear and cytoplasmic membranes lose their integrity, thus delineating a process similar to that of cell necrosis. In inflammatory processes during the activation of neutrophils, specific biochemical mechanisms determine the production of reactive oxygen species (ROS), mediated by the activation of NADPH oxidase [102]. Nicotinamide adenine dinucleotide phosphate (NADPH) oxidase promotes the cell death process with the release of NETs. As regards the specific involvement of reactive oxygen species (ROS) in the release of NETs, it occurs through a process mediated by neutrophilic elastase and myeloperoxidase. Elastase translocates from cytoplasmic granules to the nucleus triggering the degradation of chromatin through histone cleavage [56,98,102]. Instead, myeloperoxidase contributes to the decondensation of nuclear DNA [56,98,102].

Since NETs participate in various pathological processes either by inhibiting or promoting damage, NETosis in oxidative stress has been carefully reconsidered [102,103]. There is evidence to reveal that this specific program, triggered during the life of neutrophils, is not just a path to death. So much so that a second mechanism biologically classified as “vital” NETosis has been proposed [104]. During the “vital” NETosis the release of NETs is also necessary. The difference between the two processes, « death » or « vital » NETosis, lies in the nature of the precipitate stimulus, in the timing and mechanisms used to induce the release of NETs [104].

Virologists explained this specific ability that viruses have in evading the host’s immune response. This peculiar ability makes them particularly dangerous as promoters responsible for triggering the processes of NETosis [105,106,107]. Therefore, many viruses favor the production of NETs, after activating the neutrophils, with different modalities. First, the neutrophils release NETs according to the usual biological processes described above. On the other hand, neutrophils can produce antiviral agents or undertake the transition to apoptosis. It is important to underline that once the virus-induced NET production has taken place, with the constitution of double-stranded DNA complexes, histones, and granular proteins, they can circulate in an uncontrolled way. The resulting relative phenomenon is the organism’s extreme systemic response to the production of immune complexes, cytokines, and chemokines, ultimately promoting inflammation. As emerges from recent studies that have revealed cardiac complications in patients with COVID-19, NETosis induced by the virus acts on two fronts. While on one front the mechanical entrapment of the virus is observed, on the other, the inflammatory and immunological reaction triggered by the release of the NETs with the induction of potential damage is highlighted [14].

**Figure 5:**
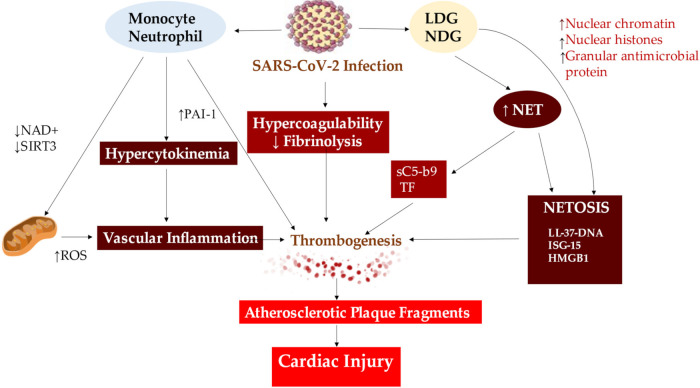
The mechanism leading to cardiac injury from NETs formation in patients with severe COVID-19 is determined by vascular inflammation, thrombogenesis and NETOSIS through the instability of the atherosclerotic plaque. Abbreviations: HMGB1, mobility group box; ISG-15; interferon-stimulated gene; LDG, low-density granulocytes; NDG, normal density granulocytes; NAD, nicotin adenin dinucleotide; ROS, reactive oxygen species; SIRT3, Sirtuin 3. Other abbreviations in previous figure. ↑, increase; ↓, decrease.

With the advent of COVID-19, NETosis activity of infected patients has garnered interest to understand whether the clinical course of the disease is as a worsening evolution or as a clinical recovery, may be conditioned by NETosis, Figure 5.

Two points should be underlined. The first concerns the fact that NETosis has been evoked as a well-defined process in the inflammatory response occurring in pulmonary diseases. In fact, evidence from bronchoalveolar lavage fluid suggested an increased level of NETs in patients with acute respiratory distress syndrome (ARDS) [108,109], as well as in patients who disclosed worse clinical condition after developing an acute respiratory failure secondary to chronic obstructive pulmonary disease (COPD) [110]. Likewise, patients with clinically severe forms of COVID-19 or who have exhibited worse progressive symptoms, sustained by the cytokine storm, develop an ARDS-like status with increased NETs [111]. The second point concerns the correlation between NETs release and thrombotic complications in COVID-19 infection, involving the arterial and venous districts [43,44]. Several studies have reported marked evidence of micro and macro thrombotic phenomena such as microangiopathy leading to pulmonary embolism [45], for which antithrombotic and/or coagulation prophylaxis was in short order initiated [43,44,45].

Histopathology from lung specimens disclosed fibrin-based blockages in the small blood vessels in COVID-19 patients’ who died [13,15,16,17,18,19]. This pathoanatomic condition mimics acquired and potentially life-threatening thrombophilia such as the antiphospholipid syndrome, in which patients develop pathogenic autoantibodies targeting phospholipids and phospholipid-binding proteins (aPL antibodies) such as prothrombin and beta 2 glycoprotein I (beta 2GPI). These antibodies undertake cell surfaces leading to the activation of endothelial cells, platelets, and neutrophils [84,112,113]. Ultimately the antibodies affect the blood-endothelium interface toward thrombosis. These aPL antibodies have recently been reported in patients who experienced COVID-19 [114,115] as well as many patients admitted to hospital with the severe form of COVID-19 who displayed NETs in their blood which may also contribute to the prothrombotic milieu [84,116].

Zuo et al. found eight types of aPL antibodies in serum samples from 172 patients who required hospitalization for COVID-19 and with a rate ranging between 30% to 52%. These aPL antibodies included anticardiolipin IgG, IgM, and IgA; anti-β2 glycoprotein I IgG, IgM, and IgA; and anti-phosphatidylserine/prothrombin (aPS/PT) IgG and IgM. Three main findings were identified in this study [46]. The first revealed that neutrophil hyperreactivity was highly dependent on superior titers of aPL antibodies, including the release of extracellular neutrophil traps (NETs), greater platelet counts, more severe respiratory disease, and clinically estimated glomerular filtration rate. Second, as was observed with the presence of a specific IgG activity in patients with antiphospholipid antibody syndrome, the presence of isolated IgG fractions that favored the release of NETs from neutrophils isolated from healthy individuals was also recorded in patients with COVID-19. Third, IgG purified from serum from COVID-19 patients was injected into two mouse models of mice causing an acceleration of venous thrombosis. The authors concluded that half of the patients seeking admission for COVID-19 experienced a transient rise in aPL antibodies; these autoantibodies are potentially pathogenic and can lead to an increase in NETs [46].

**Figure 6:**
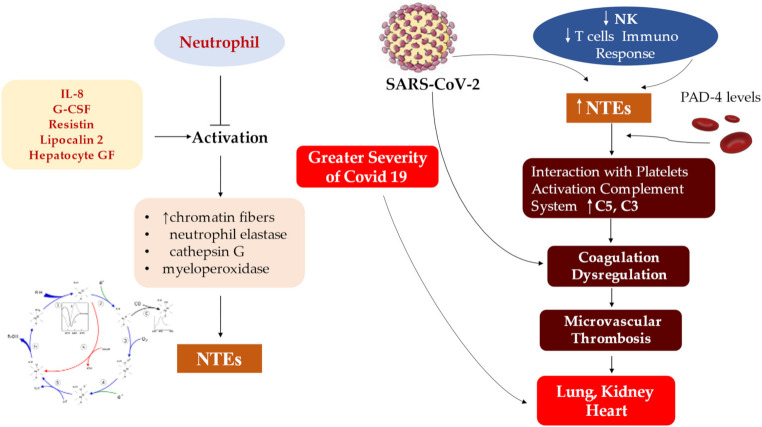
SARS-CoV-2 determines the activation of neutrophils mediated by IL-8, G-CSF, resistin, lipocalin-2, hepatocyte growth factor and NET release. The immune response of NK and T lymphocytes contributes to the formation of NETs with the increased level of a completement system (C5 and C3). The generated microvascular thrombosis leads to organ damage. Abbreviations: C, complement; GF, grow factor; IL, interleukine; NK; natural killer. Bottom left depict the biochemical reaction for the formation of NETs Other abbreviations in previous figure. ↑, increase; ↓, decrease.

These explained processes are of crucial importance as they outline the role of NETosis which appears to be substantial in all conditions characterized by venous and arterial thrombosis. Concerns related to the activity of DNAse I, an enzyme that catalyzes the digestion of NETs, and the phagocytic activity of macrophages, which profusely infiltrate the cardiac extracellular matrix in COVID-19 patients with cardiac complications [14], deserve a more in-depth evaluation. In fact, these are the two main mechanisms for regulating and self-limiting NETosis [20,21,22,23,24,25,26,27,28,29,30,31,32,33,34,35,36,37,38,39,40,41,42,43,56,57,58,59,60,61,62,63,64,65,66,67,68,69,70,71,72,73,74,75,76,77,78,79,80,81,82,83,84,85,86,87,88,89,90,91,92,93,94,95,96,97,98], show in Figure 6.

## 4. Insights into the Role of Neutrophil Extracellular Traps and Their Interference in the Heart Inflammation Process from SARS-CoV-2 Infection

Myocardial injury has a crucial role as greater provider of mortality in COVID-19. The landmark study of Zhou et al. [43] from Wuhan reported a larger percentage of mortality reaching 70% of patients hospitalized with elevated cardiac troponin I plasma levels. Acute inflammation response precipitated by SARS-CoV-2 infection is fitted together atherosclerotic plaque development and progression. The concern related to SARS-CoV-2 heart infection is directly linked to acute inflammatory stimulus, prompted by virus localization in the cardiac tissue. The development and destabilization of atherosclerotic plaque may lead to acute myocardial infarction. Several studies [1,2,13,14,18,94] have confirmed these data thus highlighting the fundamental role offered by the phenomenon of the “cytokine storm” in determining ischemic heart disease [21,23,24,68,94]. Virologists and immunologists have learned that the proinflammatory cytokines elicited by endothelial cells lead to a change in homeostatic functions with the consequent endothelial damage, the subsequent destabilization of the atherosclerotic plaque and the evolution towards thrombosis. Cytokines such as IL-1 α and IL-1β, IL-6, and TNF-α can perturb all of the protective functions of the normal endothelium so as to enhance pathological processes [21,23,24,42,68,80,81,82,94].

Specifically, IL-1 can induce its own gene expression thus leading to an amplification of the levels of IL1 that trigger the cytokine storm [14,68]. Furthermore, IL-1 promotes the expression of other proinflammatory cytokines including TNF-α. IL-1 and leukocyte migration can inspire the production of chemotactic molecules including chemokines that cause inflammatory cells to penetrate into tissues. Meanwhile, IL-1 stimulates the production of IL-6. The substantial role of IL-6, whose plasma levels are generally very low, is to promote a of immune and inflammatory responses. During acute infection, a wide kind of cells, including macrophages, B and T lymphocytes, work to determine an increase in the production of IL-6. In addition to local effects, IL-6 provides a proximal stimulus to the acute phase response [14,23,24,25,36,68,69,70,71,81,82,85].

Again, IL-6 works to support the production of fibrinogen which is the main precursor of clots and of PAI-1 which is an important inhibitor of endogenous fibrinolytic mediators. Finally, the action of IL-6 is aimed at increasing the levels of C-reactive protein, a biomarker of inflammation closely linked to COVID-19 infection. During the infection, a loss of the barrier function of the endothelium has been proved due to its activation, with a consequent increased expression of adhesion molecules such as soluble ICAM-1 (intercellular adhesion molecule 1), of soluble VCAM-1 (molecule vascular cell adhesion 1), and VWF release. The latter allows for platelet binding and TF expression which activates the coagulation system [11,34,36,37,38,39,40,86].

The evidence of NETs has been revealed in coronary thrombi from patients who exhibit STEMI and myocardial infarction as a complication of COVID-19 [14]. To date, there are no studies that have clarified precisely the intrinsic mechanism of coronary occlusion in patients with COVID-19 who develop STEMI. In this context, evidence resulting from a cohort of 55 patients who underwent primary coronary interventions for STEMI suggested that NETs play a decisive role in the pathogenesis of coronary thrombosis in COVID-19 and the onset of MI. The investigators disclosed NETs in all 5 patients with COVID-19 who received intracoronary aspirates compared to those (n = 50) without the infectious disease during the primary percutaneous coronary intervention (PCI) [14].

A relevant finding of this investigation disclosed that the median density of NET ranged at 61% (95%CI, 43–91%) and this value was remarkably higher than reported in a previous series. Rather, the investigators found that NETs reached 68% in the sampling of aspirated coronary thrombus during primary PCI from 34 patients with a median NET density reported at 19% (95% CI, 13–22%; *p* < 0.001) [117].

NETs are released by neutrophils and perform the function of trapping pathogens as they are made up of web-like structures of DNA and proteins (histones, microbicidal proteins, and oxidizing enzymes). However, dysregulation of NET function is critical in initiating and increasing inflammation and thrombosis [46,84]. Cersana et al. studied post mortem finding from a large series of pulmonary autopsy samples of COVID-19 patients’ revealing an excessive NET formation, responsible for a quickly pulmonary microvessels occlusion and severe organ damage [47]. Importantly, Blasco et al. observed an abundant amount of NET in coronary thrombi of COVID-19 patients with complicated STEMI and MI [117]. Furthermore, the burden of NET was significantly higher than that reported in a previous series of patients with STEMI but without COVID-19 infection [14,117]. From the histochemical point of view, all thrombi were constituted by conglomerates of fibrin and polymorphonuclear cells. An interesting finding suggested the absence of atherosclerotic plaque fragments that were evident in 65% of the coronary clot aggregates of the control group who experienced STEMI without infection. The preponderance of atheromatous plaque fragments supported evidence that already emerged in a previous study by the same group in 142 patients with STEMI [117].

We learned that coagulation changes associated with COVID-19 suggested the existence of a hypercoagulable state that can lead to an increase in the risk of thromboembolic complications [14,46,84,114,115,116,117]. We also know that patients with COVID-19 typically experience an increase in D-dimer concentration, a relatively lowly decrease in platelet count, and a prolongation of prothrombin time [10]. These perturbations, except for an increase in Dimer D, are not found in patients showing NET release and STEMI [14,117]. Therefore, the idea is reinforced that neutrophils and NETs play an important role in causing thrombus formation in coronary arteries of patients with COVID-19 [12,56,98]. Once again, an association between NET and unfavorable clinical outcomes after STEMI is outlined, even if no definitive results are available on the specific components of the NET measured peripherally. NET may help to define an unfavorable prognostic picture in patients with COVID-19 to which a STEMI contributes to clinical manifestations [14,117].

Lindner et al. described the presence of the viral genome in myocardial tissue from 39 autopsy samples, in which fifteen (38.5%) did not disclose SARS-CoV-2 [13]. Pneumonia occurred as the cause of death with a rate of 89.7% of individuals (n = 35) and none of the patients revealed had clinically fulminant myocarditis [13]. This finding corroborates previous evidence to support the expression of the SARS-CoV-2 spike glycoprotein selective ACE2 receptor on the surface of myocardial cells [118] as well as the substantial involvement of myocardial tissue in infection [119]. The most revealing findings highlighted by Lindner after in situ hybridization of myocardial tissue suggests the most likely localization of SARS-CoV-2 was not found in cardiomyocytes but interstitial cells or macrophages invading myocardial tissue. The investigators reported the presence of CD3+, CD45+, and CD68+. However, the cohort that exhibited the viral genome did not record an increase in mononuclear cell infiltrates into the myocardium compared to the cohort without virus. [13] Particularly, 1/3 of patients with viral load greater than 1000 copies, deemed clinically significant, revealed signs of viral replication within myocardial tissue. Investigators documented increased expression in patients with a viral load greater than 1000 copies where cytokines are currently implicated in the modulation of the inflammatory process. 16 patients had an increased expression of 6 proinflammatory genes related to cytokine production (tumor necrosis growth factor α, interferon γ, chemokine ligand 5, interleukin-6, -8, and -18) compared with 15 patients without any SARS-CoV-2 in the heart [13].

This evidence is in line with the findings of Guzik et al. who linked cytokine-induced organ dysfunction to the disease process [6]. What emerges from Lindner’s study is crucial in pointing out that patients with SARS-CoV-2 infection and viral replication did not show associated fulminant myocarditis. In fact, in this study, relevant data is offered by the lack of significant changes in the transendothelial migration of inflammatory cells in the myocardium of patients with high viral load compared to those who did not have any virus. Conversely, several studies reported a correlation between the occurrence of myocardial inflammation and evidence of clinical myocarditis. Lindner et al., therefore, offered an explanation supporting the idea that viral replication and myocarditis may not be two joint processes. Moreover, their results suggested no increased inflammatory cells in consecutive COVID-19 cases without clinical myocarditis [13].

**Figure 7:**
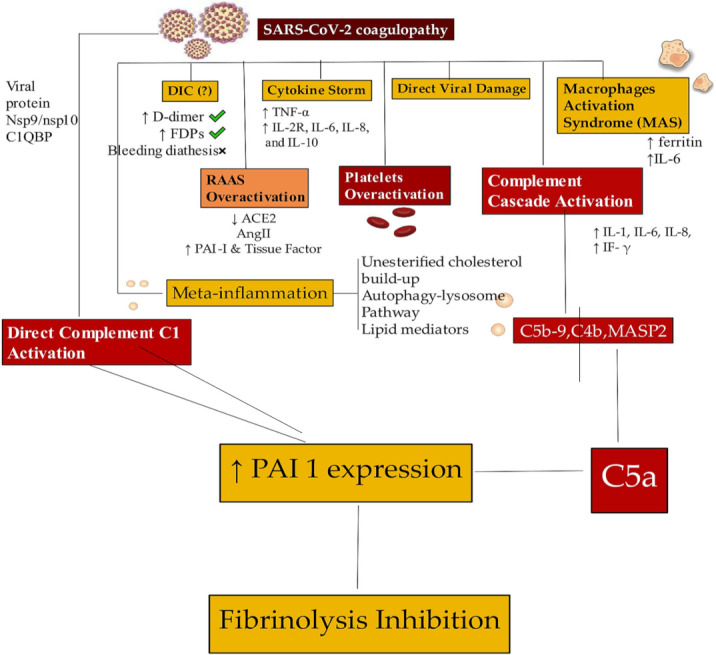
SARS-CoV-2 infection determines dysregulations in coagulation system. The coagulopathy is supported by the DIC, cytokine storm process, and direct action of the virus, inducing damage and activation of macrophages. RAAS overactivation associated with platelet and complement overactivation (direct and indirect) leads to fibrinolysis inhibition. Abbreviations are as shown in previous figures. Arrows explain the increase or decrease of relative component. ↑, increase; ↓, decrease.

The crucial point focuses on the long-term effects of the presence of the virus in myocardial tissue. Whether the presence of viral activity in the myocardium in the absence of clinical symptoms of myocarditis remains unknown. However, we know that the leukocytopenia that characterizes patients with COVID-19 could hinder the migration of activated mononuclear cells [120]. Among these cells, the scarce presence of macrophages, responsible for digesting NETs, could play a crucial role in maintaining a high NETs release level, Figure 7.

## 5. Comment: Myocardial Injury and Mortality in Patients with COVID-19

The data available from China, Italy, in the United States are in favor of a COVID-19 which occurs in a relatively mild clinical form in most of the affected individuals, but in others, COVID-19 can be life-threatening. The experiences gained in these years of the pandemic support the evidence that the individuals at the highest risk of serious illness, such as requiring intensive care hospitalization and those at the greatest risk of mortality, are older individuals, with underlying comorbidities, including cardiovascular diseases [20,23,48,49,50,51,52,53,55,121,122,123]. However, even younger adults have disclosed serious illnesses for which hospitalization and surgery were necessary with deaths in this age group reported [50,51,52,53,123].

As previously observed in other epidemiological studies focused on the clinical evolution of influenza and other diseases supported by an acute inflammatory state, patients who develop COVID-19 in the presence of diagnosed coronary artery disease and those with risk factors for atherosclerotic cardiovascular disease have an increased risk of experiencing acute coronary syndromes during the disease [124,125,126]. Established acute coronary events, similar to type 2 myocardial infarction, could be related to the significant increase in myocardial demand directly related to infection that can lead to myocardial damage or infarction [127]. However, there is the possibility that an uncontrolled increase in the levels of circulating cytokines released during intense systemic inflammatory activity can lead to instability or even rupture of the atherosclerotic plaque. Another possible comparison with COVID-19 patients concerns patients with heart failure who can manifest an evolution towards haemodynamic decompensation during stressful conditions related to serious infectious diseases [50,51,52,53,123].

What emerged from the published reports found that patients with underlying cardiovascular disease, which are more prevalent in the elderly, are more prone to higher risks of adverse outcomes and death during the more aggressive forms of COVID-19 sustained by severe inflammatory states, compared to younger patients. It should be noted that similar to the Middle East Respiratory Syndrome coronavirus outbreak, acute/fulminant myocarditis associated with heart failure has been described in SARS-CoV-2 as well.

Two independent Chinese reports [1,2] describing hospital series from Wuhan, have corroborated these concepts while providing new evidence regarding the incidence and consequences of myocardial lesions associated with SARS-CoV-2. In the first study [1] investigators analyzed a cohort of 416 hospitalized patients with COVID-19, using the highly sensitive reverse transcriptase-polymerase chain reaction technique, confirmed that 19.7% (n = 82) of patients revealed myocardial damage from the increase in troponin I (TnI) levels. Patients with myocardial damage had a hospital stay with a significantly higher mortality rate of 51.2% (42 of 82) than 4.5% without myocardial damage (15 of 335). Furthermore, in patients with myocardial damage, higher levels of TnI elevation were associated with higher mortality rates.

The second report [2] supports the above with 11 in a cohort of 187 hospitalized patients with laboratory confirmed COVID-19, of which 27.8% (n = 52) revealed myocardial damage noted by elevated troponin T (TnT) levels, providing additional novel insights concerning levels of C-reactive protein and N-terminal pro-B-type natriuretic peptide (NT-proBNP). First, investigators pointed to a rate of in-hospital mortality of 59.6% (31 out of 52) in patients with high TnT levels compared to 8.9% (12 out of 135) in those with normal TnT levels. Other relevant evidence supported that the highest mortality rates of 69.4% (25 out of 36) were recorded in individuals with elevated TnT levels where the underlying cardiovascular disease was noted. Another crucial point suggested that mortality rates were lower in patients with high TnT levels without prior cardiovascular history. Second, patients with known cardiovascular disease without elevation of TnT levels disclosed a mortality rate that was relatively favorable despite a mortality rate of 13.3% (4 of 30). Third, TnT levels were significantly associated with levels of C-reactive protein and N-terminal pro-B-type natriuretic peptide (NT-proBNP), thus relating myocardial damage to the severity of the inflammatory state and ventricular dysfunction. Both TnT and NT-proBNP levels recorded progressive serial increases during hospitalization in patients with progressively deteriorating clinical courses. Conversely, patients with a less severe form of the disease and more favorable outcomes with lower levels of these biomarkers [2].

The studies of Shi et al. and Guo et al. carried out at the beginning of the pandemic on the Wuhan population have offered us a picture with substantially similar characteristics in patients with COVID-19 and elevated levels of TnI or TnT, who develop myocardial damage with adverse outcomes [1,2]. Patients at risk of myocardial damage have more advanced age and higher comorbidities such as the increased prevalence of hypertension, coronary artery disease, heart failure, and diabetes compared to the cohorts with normal levels of TnI or TnT. Evidence of more severe systemic inflammation is indisputable in patients with myocardial damage, including substantial increases in PMNs, higher levels of C-reactive protein and procalcitonin as well as high levels of other myocardial biomarkers injury and stress, such as elevated creatine kinase, myoglobin, and NT-proBNP. A finding that emerges in patients with COVID-19 and associated myocardial injury concerns the presence of a greater acuity of the disease, with a higher incidence of acute respiratory distress syndrome and more frequent necessitation of mechanical ventilatory support compared to those without myocardial damage. Therefore, the picture that arises from these two studies, confirmed by other reports based on cardiac autopsy and PCI performed in patients with COVID-19, is consistent with the history of patients who experienced the disease. The picture offers older patients who have contracted SARS-CoV-2 with pre-existing cardiovascular comorbidities and diabetes who are most prone to developing the disease with greater clinical acuity. These individuals have an associated increased risk of developing myocardial damage and a significantly higher short-term mortality rate [1,2].

The first report carried out on the Wuhan population represents a window that opens to further evaluations. For example, Yang and Zin discussed the relationship between cardiovascular complications during the COVID-19 outbreak in China and the underlying cardiovascular outbreak that has been studied in China for decades [128]. Investigators agree with many recent observations that the occurrence of pre-existing cardiovascular comorbidity leads to the most adverse complications of COVID-19, including death [128]. However, it is important to point out that only with subsequent reports, highlighting systemic inflammation and an uncontrolled coagulopathy in COVID-19, was a more complete explanation offered those serious infections can destabilize patients with coronary artery disease or heart failure [49,50,51,52,53,122,123].

The important association between myocardial damage and adverse outcomes has focused its attention on possible complementary mechanisms such as intense systemic inflammatory stimuli that favors greater oxygen consumption resulting in demand ischemia which evolves into myocardial damage or plaque rupture stimulated by SARS-CoV-2 behaves similarly to other coronaviruses as it can elicit the intense release of multiple cytokine and chemokines [23,24,25,26,27,28,29,30,31,32,33,34,35,36,37,38,39,40,41,42,43,44,45,46,47,48,49,50,51,52,53,69,70,71,72,73,74,75,76,77,78,79,80,81,82,83,84,85,86,87,88,89,90,91,92,93,94,95,96,97,98,99,100,101,102,103,104,105,106,107,108,109,110,111,112,113,114,115,116,117,118,119,120,121,122,123,124,125,126,127,128]. This stage is decisive not only in favoring vascular inflammation, plaque instability, and inflammation of the myocardium but also in triggering the release of NETs.

In some patients with or without pre-existing cardiovascular comorbidities, myocarditis may occur as COVID-19 coupled myocardial damage [129]. Again, after the well-documented case of acute myocarditis following a respiratory infection associated with COVID-19 in a 53-year-old Italian woman, several studies have documented that direct viral infection of the myocardium is another possible causal pathway of myocardial damage [5]. However, in cardiac autopsies, the virus was found in interstitial myocardial tissue without the presence of replication in myocardial cells lacking unequivocal myocarditis [13].

We have learned the existence of the affinity of SARS-CoV-2 to the host angiotensin-converting enzyme 2 receptor [1,2,128], which has been shown previously for other coronaviruses [119], raising the possibility of direct viral infection of vascular endothelium and myocardium. Although the cardiovascular complications of acute COVID-19 disease are well described, the post-acute cardiovascular manifestations that characterize COVID-19 have not yet been fully elucidated. Al-Aly et al. and Xie et al. using the national health care database of the United States Department of Veterans Affairs created a cohort of 153,760 individuals with COVID-19, to which two groups of control cohorts with 5,637,647 (contemporary controls) and 5,859,411 (historical controls) were added [32,54]. The authors using this large population estimated risks and 1-year charges of a set of pre-specified cardiovascular outcomes. Interestingly Xie et al. noted that beyond the first 30 days of the infectious incident, patients with COVID-19 had an increased risk of cardiovascular disease-related events affecting several categories, including cerebrovascular disorders, arrhythmias, ischemic and non-ischemic heart disease, pericarditis, myocarditis, heart failure, and thromboembolic disease [54].

**Figure 8:**
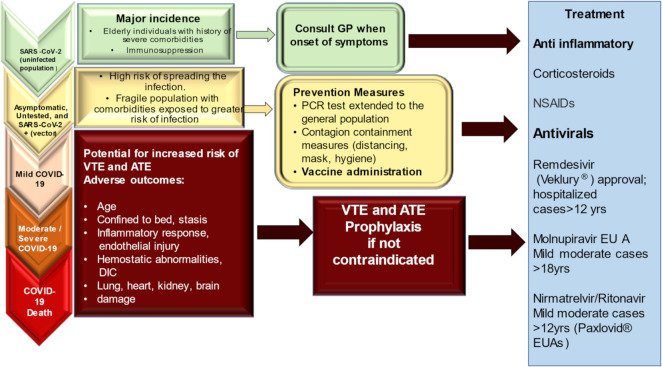
The infection from SARS-CoV-2 caused a variability in the manifestation of the disease. This explains the different population rates of infection and the distinct mortality rates of manifest cases in various regions and countries. Inflammatory response, increased age, and bed rest, which are most frequently seen in severe coronavirus disease 2019 (COVID-19), may contribute to thrombosis and adverse events resulting from multiorgan involvement. FDA timeline of antivirals approval and EUAs. Veklury^®^ EUA was formalized in January 2020. Its definitive approval occurred in October, 2020. Molnupiravir and Paxlovid^®^ EUAs followed in December 2021. Abbreviations: ATE, arterial thromboembolism.; COVID-19, coronavirus disease 2019; DIC, disseminated intravascular coagulation; EUA: Emergency Use Authorization; FDA: Food and Drug Administration; NSAIDs, non-steroid anti-inflammatory drugs; SARS-CoV-2, severe acute respiratory syndrome-coronavirus-2; VTE, venous thromboembolism.

The results reported by Xie et al. offer a crucial explanation of how these risks and charges were evident even among individuals for whom hospitalization was not required during the acute phase of the infection. The risk of developing a cardiovascular complication gradually increased based on the care setting in which patients were treated during the acute phase. The risk was lower in non-hospitalized patients, followed by hospitalized patients, and higher in ICU patients. The findings described in the report by Xie et al. support evidence that both the 1-year risk and burden of cardiovascular disease in acute COVID-19 survivors were considerable. COVID-19 is a disease with a high social impact and particular attention to the care pathways of those who survive the acute episode of COVID-19 is required. Attention to cardiovascular health and disease should be included among these [54], Figure 8.

## 6. Future Direction

New challenges await scientific community and among these the rewiring of granulopoiesis can offer a therapeutically relevant implication for trained immunity. In fact, a crucial role in successfully coping with SARS-CoV-2 infection can be offered by the trained innate immunity that is induced through the modulation of mature myeloid cells or their bone marrow progenitors. The bacillus of the Calmette-Guérin tuberculosis vaccine (BCG) has been shown to protect against certain heterologous infections through a process known as trained immunity. This type of immunity is probably achieved through the induction of innate nonspecific immune memory in monocytes and natural killer (NK) cells. Two recent independent studies revealed that induction of trained immunity is associated with a tendency to granulopoiesis in bone marrow hematopoietic progenitor cells [130,131,132].

The first study found that BCG vaccination of healthy humans induced long-lasting changes in the neutrophil phenotype, characterized by increased expression of activation markers and antimicrobial function. Evidence has suggested that enhanced human neutrophil function persists for at least 3 months after vaccination and is associated with genome-wide epigenetic modifications in histone 3 lysine 4 trimethylation [130].

In the second study promising evidence emerged on improving antitumor immunity that can be improved through the induction of trained immunity. Mouse models pre-treated with β-glucan, a prototype of fungal-derived trained immunity agonist, revealed a substantial decrease in tumor growth. The antitumor effect of trained immunity induced by β-glucan, is associated with the transcriptomic and epigenetic rewiring of granulopoiesis and the reprogramming of neutrophils towards an antitumor phenotype. This process requires signaling of type I interferon, regardless of adaptive immunity in the host. Adoptive transfer of neutrophils from β-glucan-trained mice to untreated recipients suppressed tumor growth by ROS-dependent action [131,132].

## 7. Conclusions

The cardiovascular implications of the COVID-19 pandemic have caused significant morbidity and mortality. The process of understanding the mechanism for the manifestation of these adverse outcomes is crucial to permit treatment and management options for these patients. The adverse cardiovascular outcomes manifest in several different manners from demand-induced ischaemia, coronary obstruction, and direct myocardial infiltration alongside others. The long-term effects of this pandemic, however, remain uncertain and require ongoing monitoring and research as the endemic phase of the disease is embraced. Functional reprogramming of neutrophils by inducing trained immunity could offer original therapeutic strategies in clinical conditions that could benefit from modulation of neutrophil effector function.

## Figures and Tables

**Table 1:** Characteristics of the included studies.

Author/Year	Study Period	Total Number	COVID-19 Study Design	Hospitals/Centers	Type
Shi (2020) [1] *JAMA*	20 January 2020 to 10 February 2020	416	Clinical, laboratory, radiological, and treatment	Single Center Wuhan, China	Prospective
Guo (2020) [2] *JAMA Cardiology*	20 January 2020 to 10 February 2020	187	Clinical laboratory comorbidities, and treatments	Single Center Wuhan, China	Observational
Szekely (2020) [3] *Circulation*	21 March 2020 to 16 April 2020	100	Echocardiographic	Single Center Israel	Prospective
Lala (2020) [4] *JACC*	27 February 2020 to 12 April 2020	506	Clinical, laboratory, Echocardiographic	Single Center NYC, NY, USA	Prospective
Escher (2020) [7] *ESC Heart Fail*	3 February 2020 to 26 March 2020	104	Endomyocardial biopsies	Multicenter Germany	Prospective
Lindner (2020) [13] *JAMA Cardiology*	8 April 2020 to 18 April 2020	39	Autopsy	Multicenter Germany	Prospective
Blasco (2020) [14] *JAMA Cardiology*	24 March 2020 to 11 April 2020	55	PCI/Coronary aspirates, NETs	Single Center Spain	Prospective
Ackermann (2020) [15] *NEJM*	2019 ^†^ 2009 ^††^	24	Pulmonary autopsy/Immune profiling	Multicenter Germany/USA	Comparative study
Bryce (2021) [16] *Mod. Pathol.*	20 March 2020 to 23 June 2020	100	Pulmonary autopsy/Immune profiling	Single Center NYC, NY, USA	Prospective
Schaefer (2020) [17] *Mod. Pathol.*	April 2020	7	Pulmonary autopsy/Immune profiling	Single Center Boston, MA, USA	Observational
Varga (2020) [18] *Lancet*	« « «	3	Autopsy/Immune profiling	Multicenter Switzerland/USA	Observational
Delorey (2021) [19] *Nature*	« « «	17	Autopsy/Immune profiling	Multicenter USA	Comparative study
Wang (2020) [20] *JAMA*	1 January 2020 to 28 January 2020	138	Clinical, laboratory, radiological, and treatment	Single Center Wuhan, China	Observational
Lucas (2020) [21] *Nature*	18 March 2020 to 27 May 2020	113	Immune profiling	Multicenter USA	Observational
Yang (2020) [22] *J Allergy Clin. Immunol.*	« « «	50	Immune profiling	Multicenter China	Observational
Huang (2020) [23] *Lancet*	16 December 2019 to 2 January 2020	41	Immune profiling	Multicenter China	Observational
Liu (2020) [24] *J. Infect.*	11 January 2020 to 29 January 2020	245	Immune profiling	Multicenter China/UK	Observational
Rodriguez (2021) [25] *J. Exp. Med.*	« « «	124	Autopsy/Immune profiling	Multicenter Brasil	Observational
Burkhard-Koren (2021) [26] *J. Pathol. Clin. Res.*	May 1918 to April 1919 2009–2020 Until 2020	411	Autopsy/Immune profiling	Single center Switzerland	Comparative study
Sang (2021) [27] *Cardiovasc. Pathol.*	Until 2021	50	Autopsy/Immune profiling	Single Center Birmingham, AL, USA	Observational
Melms (2021) [28] *Nature*	Until 2021	26	Autopsy/Immune profiling	Multicenter USA	Comparative study
Qin (2020) [29] *Clin. Infect. Dis.*	10 January 2020 to 12 February 2020	452	Immune profiling	Single Center Wuhan, China	Observational
Wilk (2020) [30] *Nat. Med.*	March–April 2020	7	Immune profiling	Single Center Stanford, CA, USA	Prospective
Wang (2020) [31] *Front. Immunol.*	23 January 2020 to 15 March 2020	55	Immune profiling/NETs	Multicenter China/Germany	Observational
Al-Aly (2021) [32] *Nature*	Until 2021	73,435	Clinical, laboratory	Single Center Saint Louis, MO, USA	Observational
Xie (2020) [33] *Br. Med. J.*	1 January 2017 to 31 January 2019 2 January 2020 to 17 June 2020	16,317	Clinical, laboratory	Single Center Saint Louis, MO, USA	Comparative study
Piazza (2020) [34] *JACC*	13 March 2020 to 3 April 2020	1114	Clinical Thromboembolic Complication	Single Center Boston, MA, USA	Observational
Zhang (2020) [35] *J. Thromb. Thrombolysis*	23 February 2020 to 3 March 2020	12	Clinical Thromboembolic Complication	Multicenter China	Prospective
Liu (2020) [36] *J. Transl. Med*.	1 February 2020 to 24 February 2020	61	Immune profiling	Single Center Beijing, China	Prospective
Fu (2020) [37] *Thromb. Res.*	20 January 2020 to 20 February 2020	75	Immune profiling Thromboembolic Complication	Single Center Suzhou, China	Comparative study
Webb (2020) [38] *Lancet Rheumatol.*	13 March 2020 to 5 May 2020	299	Immune profiling	Multicenter USA	Observational
Ye (2020) [39] *Respir.* *Res.*	1 January 2020 to 16 March 2020	349	Immune profiling Thromboembolic Complication	Multicenter China	Prospective
Tatum (2020) [40] *Shock*	Until 2021	125	Immune profiling	Multicenter USA	Multicenter Prospective Registry
Yang (2020) [41] *Int. Immunopharmacol.*	Until 20 February 2020	93	Immune profiling	Multicenter China	Observational
Wang (2020) [42] *Int. Immunopharmacol.*	15 January 2020 to 2 March 2020	95	Immune profiling	Single Center Wuhan, China	Observational
Zhou (2020) [43] *Lancet*	29 December 2019 to 30 January 2020	191	Clinical, laboratory, radiological, and treatment	Multicenter China	Observational
Klok (2020) [44] *Thromb. Res.*	7 March 2020 to 5 April 2020	184	Thromboembolic Complication	Multicenter Netherlands	Prospective
Tang (2020) [45] *J. Thromb. Haemost.*	1 January 2020 to 13 February 2020	448	Thromboembolic Complication	Single Center Wuhan, China	Observational
Zuo (2020) [46] *Sci. Transl. Med.*	« « « «	172	Immune profiling Thromboembolic Complication/NETs	Multicenter China/USA	Prospective
Carsana (2020) [47] *Lancet Infect. Dis.*	29 February 2020 to 24 March 2020	38	Autopsy/Immune profiling	Multicenter Italy	Observational
Chen (2020) [48] *Lancet*	1 January 2020 to 20 January 2020	99	Clinical, laboratory, radiological, and treatment	Multicenter China	Observational
Guan (2020) [49] *NEJM*	11 December 2019 to 29 January 2020	1099	Clinical, laboratory, radiological, and treatment	Multicenter China	Observational
COVIDSurg Collaborative (2022) [50] *Anaesthesia*	10 January 2020 to 30 January 2020	128,013	Thromboembolic Complication	Multicenter	Prospective
COVIDSurg Collaborative (2021) [51] *Anaesthesia*	10 January 2020 to 30 January 2020	96,454	Clinical	Multicenter	Prospective
COVIDSurg Collaborative (2021) [52] *Br. J. Surg.*	10 January 2020 to 30 January 2020	56,589	Clinical/Vaccine effectiveness	Multicenter	Prospective
COVIDSurg Collaborative (2021) [53] *Anaesthesia*	10 January 2020 to 30 January 2020	140,231	Clinical	Multicenter	Prospective
Xie (2022) [54] *Nat. Med.*	1 March 2020 to 15 January 2021	153,760	Clinical	Multicenter USA	Observational

Abbreviations: †, it refers to the flu pandemic; ††, it refers to the flu pandemic.

**Table 2:** Prisma checklist. n/a = not application.

Section and Topic	Item #	Checklist Item	Location Where Item Is Reported
TITLE			
Title	1	Identify the report as a systematic review.	Title and introduction
ABSTRACT			
Abstract	2	See the PRISMA 2020 for Abstracts checklist.	Abstract
INTRODUCTION			
Rationale	3	Describe the rationale for the review in the context of existing knowledge.	Introduction
Objectives	4	Provide an explicit statement of the objective(s) or question(s) the review addresses.	Introduction
METHODS			
Eligibility criteria	5	Specify the inclusion and exclusion criteria for the review and how studies were grouped for the syntheses.	Methods
Information sources	6	Specify all databases, registers, websites, organisations, reference lists and other sources searched or consulted to identify studies. Specify the date when each source was last searched or consulted.	Methods/PRISMA statement
Search strategy	7	Present the full search strategies for all databases, registers and websites, including any filters and limits used.	Methods
Selection process	8	Specify the methods used to decide whether a study met the inclusion criteria of the review, including how many reviewers screened each record and each report retrieved, whether they worked independently, and if applicable, details of automation tools used in the process.	Methods
Data collection process	9	Specify the methods used to collect data from reports, including how many reviewers collected data from each report, whether they worked independently, any processes for obtaining or confirming data from study investigators, and if applicable, details of automation tools used in the process.	Methods
Data items	10a	List and define all outcomes for which data were sought. Specify whether all results that were compatible with each outcome domain in each study were sought (e.g., for all measures, time points, analyses), and if not, the methods used to decide which results to collect.	Methods
	10b	List and define all other variables for which data were sought (e.g. participant and intervention characteristics, funding sources). Describe any assumptions made about any missing or unclear information.	Methods
Study risk of bias assessment	11	Specify the methods used to assess risk of bias in the included studies, including details of the tool(s) used, how many reviewers assessed each study and whether they worked independently, and if applicable, details of automation tools used in the process.	n/a
Effect measures	12	Specify for each outcome the effect measure(s) (e.g., risk ratio, mean difference) used in the synthesis or presentation of results.	n/a
Synthesis methods	13a	Describe the processes used to decide which studies were eligible for each synthesis (e.g., tabulating the study intervention characteristics and comparing against the planned groups for each synthesis (item #5)).	Methods
	13b	Describe any methods required to prepare the data for presentation or synthesis, such as handling of missing summary statistics, or data conversions.	n/a
	13c	Describe any methods used to tabulate or visually display results of individual studies and syntheses.	Methods
	13d	Describe any methods used to synthesize results and provide a rationale for the choice(s). If meta-analysis was performed, describe the model(s), method(s) to identify the presence and extent of statistical heterogeneity, and software package(s) used.	n/a
	13e	Describe any methods used to explore possible causes of heterogeneity among study results (e.g., subgroup analysis, meta-regression).	n/a
	13f	Describe any sensitivity analyses conducted to assess robustness of the synthesized results.	n/a
Reporting bias assessment	14	Describe any methods used to assess risk of bias due to missing results in a synthesis (arising from reporting biases).	n/a
Certainty assessment	15	Describe any methods used to assess certainty (or confidence) in the body of evidence for an outcome.	n/a
RESULTS			
Study selection	16a	Describe the results of the search and selection process, from the number of records identified in the search to the number of studies included in the review, ideally using a flow diagram.	Prisma diagram
	16b	Cite studies that might appear to meet the inclusion criteria, but which were excluded, and explain why they were excluded.	Prisma diagram
Study characteristics	17	Cite each included study and present its characteristics.	Table 1
Risk of bias in studies	18	Present assessments of risk of bias for each included study.	n/a
Results of individual studies	19	For all outcomes, present, for each study: (a) summary statistics for each group (where appropriate) and (b) an effect estimate and its precision (e.g., confidence/credible interval), ideally using structured tables or plots.	n/a
Results of syntheses	20a	For each synthesis, briefly summarise the characteristics and risk of bias among contributing studies.	n/a
	20b	Present results of all statistical syntheses conducted. If meta-analysis was carried out, present for each the summary estimate and its precision (e.g., confidence/credible interval) and measures of statistical heterogeneity. If comparing groups, describe the direction of the effect.	Table 1
	20c	Present results of all investigations of possible causes of heterogeneity among study results.	n/a
	20d	Present results of all sensitivity analyses conducted to assess the robustness of the synthesized results.	n/a
Reporting biases	21	Present assessments of risk of bias due to missing results (arising from reporting biases) for each synthesis assessed.	n/a
Certainty of evidence	22	Present assessments of certainty (or confidence) in the body of evidence for each outcome assessed.	n/a
DISCUSSION			
Discussion	23a	Provide a general interpretation of the results in the context of other evidence.	3.2
	23b	Discuss any limitations of the evidence included in the review.	n/a
	23c	Discuss any limitations of the review processes used.	n/a
	23d	Discuss implications of the results for practice, policy, and future research.	3.2
OTHER INFORMATION			
Registration and protocol	24a	Provide registration information for the review, including register name and registration number, or state that the review was not registered.	Methods
	24b	Indicate where the review protocol can be accessed, or state that a protocol was not prepared.	Methods
	24c	Describe and explain any amendments to information provided at registration or in the protocol.	n/a
Support	25	Describe sources of financial or non-financial support for the review, and the role of the funders or sponsors in the review.	Methods
Competing interests	26	Declare any competing interests of review authors.	Methods
Availability of data, code and other materials	27	Report which of the following are publicly available and where they can be found: template data collection forms; data extracted from included studies; data used for all analyses; analytic code; any other materials used in the review.	n/a

## Data Availability

Not applicable.

## Abbreviations

ACE1	angiotensin I-converting enzyme
ACE2	Angiotensin-Converting Enzyme 2
ACEi	ACE–inhibitors
aPL	antiphospholipid
aPS/PT Ab	anti-phosphatidylserine/prothrombin autoantibodies
APS	antiphospholipid syndrome
ARDS	acute respiratory distress syndrome
AT1R	Angiotensin Type 1 Receptor
C	complement
CCL	chemokine ligand
COPD	Chronic Obstructive Pulmonary Disease
COVID-19	Coronavirus disease-2019
CRP	C-reactive protein
CXCL	chemokine ligand
CXCL8	Interleukine 8
CVD	cardiovascular disease
DAD	diffuse alveolar damage
DIC	disseminated intravascular coagulation
ECM	extracellular matrix
FDP	fibrinogen derived peptides
G-CSF	granulocytes colony-stimulating factor
GF	grow factor
GM-CSF	granulocyte-macrophage colony stimulating factor
HLA-DR	human leucocyte antigen- related D
ICAM-1	intercellular adhesion molecule 1
ICU	intensive care unit
IFN	interféron
IL	interleukine
IP-10	interferon-gamma-induced protein
IRF	interferon regulatory factors
ISG-15	interferon stimulated gene 15
LDG	low-density granulocytes
mAb	monoclonal antibody
MASP2	mannose-binding protein associated serine protease 2
MAS	macrophage activation syndrome.
MCP-1	monocyte chemoattractant protein-1
M-CSF	macrophage colony-stimulating factor
MIP 1	macrophage inflammatory protein 1
NADPH	Nicotinamide adenine dinucleotide phosphate
NAR	neutrophil count to albumin ratio
NCD4LR	neutrophil/CD4 + lymphocyte index
NCT	negative conversion time
NDG	normal density granulocytes
NETs	neutrophil extracellular traps.
NHBE	human bronchial epithelial cell
NLR	neutrophil-to-lymphocyte ratio
NLRP3	NOD-like receptor family, pyrin domain containing 3
PAD	peptidyl arginine deaminase
PAI	platelet activator inhibitor
PBMC	peripheral blood immune cells
PMN	polymorphonuclear
RAAS	renin-angiotensin. -aldosterone system
RE	response element
ROS	reactive oxygen species
SARS-CoV-2	severe acute respiratory syndrome-coronavirus-2
Sirtuin 3	SIRT3,
STEMI	ST elevation myocardial infarction
TF	tissue factor
TFPI	tissue factor pathway inhibitor
TGF beta-2	transforming grow factor beta-2
Th	T-helper
TNF	tumor necrosis factor
TRAP	thrombin receptor-activating peptide
β2GPI	β2 I glycoprotein
